# Supplementary material for: Hydroculture Cultivation of Strawberries as Potential Reference Material for Microcystin Analysis: Approaches and Pitfalls
Source: Toxins (Basel). 2025 Jun 6;17(6):285. doi: 10.3390/toxins17060285 (PMC12197505; doi:10.3390/toxins17060285)
Supplement: Supplementary file 1 [file toxins-17-00285-s001.zip › toxins-3643836-supplementary.pdf]

Supplementary materials: Hydroculture Cultivation of Strawberries as Potential Reference Material for Microcystin Analysis: Approaches and Pitfalls

Wannes Hugo R. Van Hassel, Benoît Guillaume and Julien Masquelier

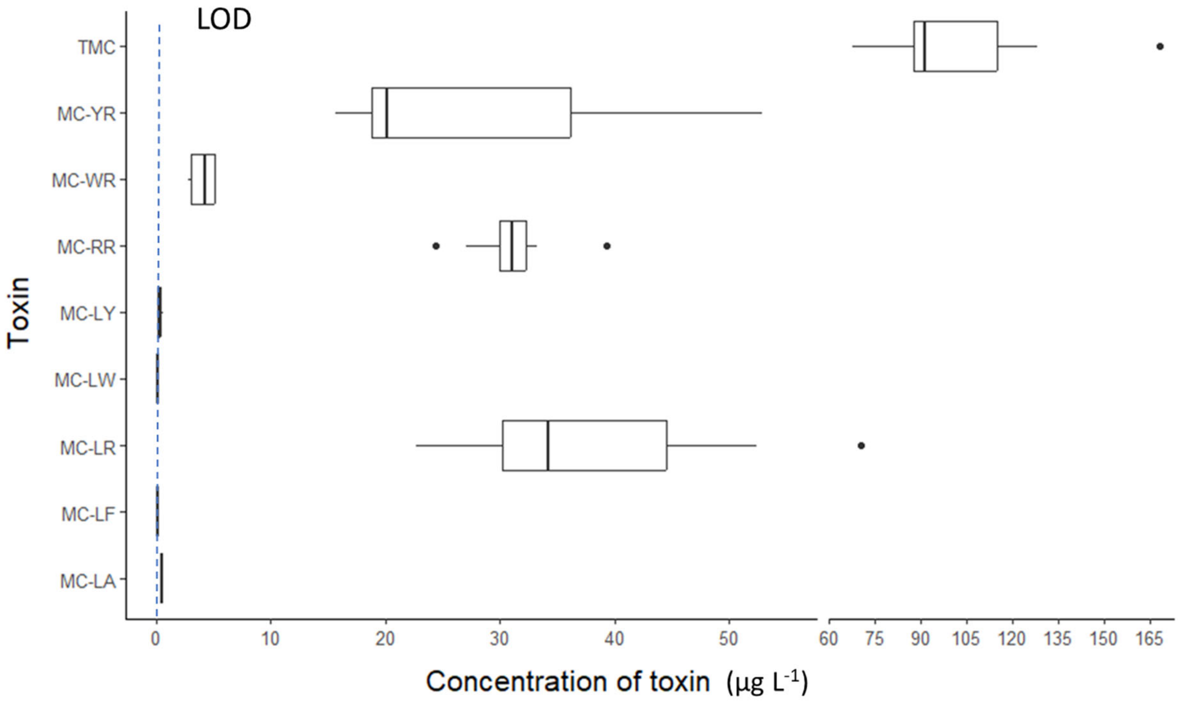

Figure S1. Microcystin congener concentrations for the natural microcystin congener mix in Hoagland solution, as well as the corresponding total measured microcystins (TMC). Toxins detected below the Limit of Quantification (LOQ) (0.5 µg L^{-1}) are represented at the level of the Limit of Detection (LOD) (0.1 µg L^{-1}). The LOD is illustrated by the blue dotted line.

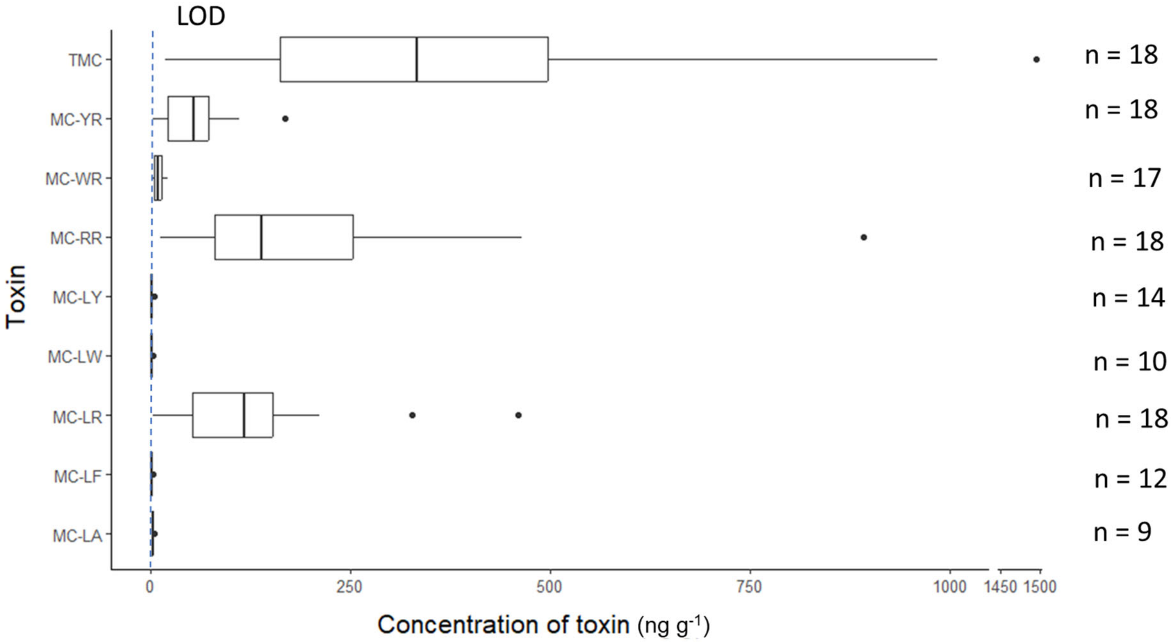

Figure S2. Microcystin congener concentrations for the natural microcystin congener mix in roots, as well as the corresponding total measured microcystins (TMC). Toxins detected below the Limit of Quantification (LOQ) (1 ng

g^{-1}) are represented at the level of the Limit of Detection (LOD) (0.6 ng g^{-1}). The LOD is illustrated by the blue dotted line. 'n' is the total number of samples in which MCs were detected.

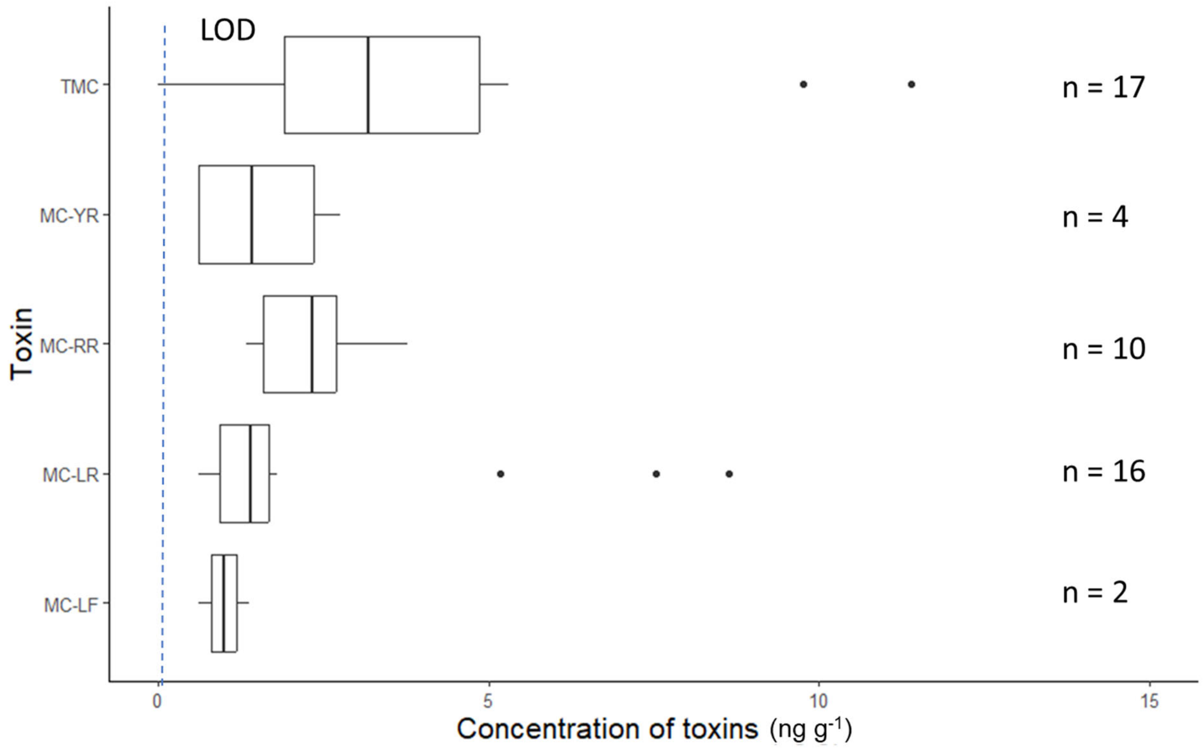

Figure S3. Microcystin congener concentrations for the natural microcystin congener mix in greens, as well as the corresponding total measured microcystins (TMCs). Toxins detected below the Limit of Quantification (LOQ) (1 ng g^{-1}) are represented at the level of the Limit of Detection (LOD) (0.6 ng g^{-1}). The LOD is illustrated by the blue dotted line. 'n' is the total number of samples in which MCs were detected.

Table S1. Microcystin congeners concentration (µg L^{-1}) observed in Hoagland solution after contamination with either freeze-dried cyanobacterial biomass (NM) or pure MC-LR.

Concentration of microcystin congeners in Hoagland samples (µg L^{-1})											
Samples	cultivation condition	MC-RR	NOD	MC-LA	MC-LF	MC-LR	MC-LY	MC-LW	MC-YR	MC-WR	Total
STR_T_H_H_CT 1606	NM	31.3	<LOD	0.5	<LOQ	48.4	<LOQ	<LOQ	37.9	2.8	121.3
STR_T_H_H_CT 0205	NM	39.3	<LOD	0.6	<LOQ	70.3	0.6	<LOQ	52.9	4.1	168.0
STR_T_H_H_CT 3005	NM	31.1	<LOD	0.6	<LOQ	52.3	<LOQ	<LOQ	41.0	3.1	128.3
STR_T_H_H_CT 2605	NM	30.4	<LOD	0.6	<LOD	43.1	<LOQ	<LOQ	35.5	3.1	112.9
STR_T_H_H_CT1906	NM	27.1	<LOD	<LOD	<LOD	39.8	<LOQ	<LOQ	30.8	2.8	100.7
240530_H_CT	NM	33.3	<LOD	0.5	<LOQ	33.0	<LOQ	<LOQ	19.9	4.9	92.2
240607_H_CT	NM	29.0	<LOD	0.5	<LOQ	28.3	<LOQ	<LOQ	17.1	4.5	79.8
240614_H_CT	NM	30.3	<LOD	0.5	<LOQ	35.4	<LOQ	<LOQ	17.7	5.2	89.7
240529_H_CT	NM	32.0	<LOD	0.5	<LOQ	30.4	<LOQ	<LOQ	19.6	5.3	88.4
240603_H_CT	NM	33.0	<LOD	0.5	<LOQ	31.4	<LOQ	<LOQ	20.4	5.1	90.9
240616_H_CT	NM	24.4	<LOD	<LOQ	<LOQ	22.7	<LOQ	<LOQ	15.7	4.0	67.6
240619_H_CT	NM	31.0	<LOD	0.5	<LOQ	29.5	<LOQ	<LOQ	19.2	5.3	86.0
STR_T_H_H_MC 1606	MC-LR	<LOD	<LOD	<LOD	<LOD	3.6	<LOD	<LOD	<LOD	<LOD	3.6
STR_T_H_H_MC 0205	MC-LR	<LOD	<LOD	<LOD	<LOD	3.4	<LOD	<LOD	<LOD	<LOD	3.4
STR_T_H_H_MC 3005	MC-LR	<LOD	<LOD	<LOD	<LOD	2.8	<LOD	<LOD	<LOD	<LOD	2.8
STR_T_H_H_MC 2605	MC-LR	<LOD	<LOD	<LOD	<LOD	1.8	<LOD	<LOD	<LOD	<LOD	1.8
STR_T_H_H_MC 1906	MC-LR	<LOD	<LOD	<LOD	<LOD	3.7	<LOD	<LOD	<LOD	<LOD	3.7
240526_H_MC	MC-LR	<LOD	<LOD	<LOD	<LOD	1.2	<LOD	<LOD	<LOD	<LOD	1.2
240530_H_MC	MC-LR	<LOD	<LOD	<LOD	<LOD	2.1	<LOD	<LOD	<LOD	<LOD	2.1
240607_H_MC	MC-LR	<LOD	<LOD	<LOD	<LOD	40.1	<LOD	<LOD	<LOQ	<LOD	40.2
240616_H_MC	MC-LR	<LOD	<LOD	<LOD	<LOD	2.1	<LOD	<LOD	<LOD	<LOD	2.1
240619_H_MC	MC-LR	<LOD	<LOD	<LOD	<LOD	2.7	<LOD	<LOD	<LOD	<LOD	2.7
240614_H_MC	MC-LR	<LOD	<LOD	<LOD	<LOD	28.0	<LOD	<LOD	<LOD	<LOD	28.0
240529_H_MC	MC-LR	<LOD	<LOD	<LOD	<LOD	43.0	<LOD	<LOD	<LOQ	<LOD	43.1
240603_H_MC	MC-LR	<LOD	<LOD	<LOD	<LOD	46.3	<LOD	<LOD	<LOQ	<LOD	46.4
240617_H_MC	MC-LR	<LOD	<LOD	<LOD	<LOD	42.2	<LOD	<LOD	<LOD	<LOD	42.2

Table S2. Microcystin congeners concentration (ng g^{-1}) observed in the roots of strawberry plants after contamination with Hoagland solution fortified with either freeze-dried cyanobacterial biomass (NM) or pure MC-LR.

Microcystin congener concentrations in root samples (ng g^{-1})											
Samples	Cultivation condition	MC-RR	NOD	MC-LA	MC-LF	MC-LR	MC-LY	MC-LW	MC-YR	MC-WR	Total MCs
2024_STR_T_R_H_CT_1	NM	464.8	<LOD	3.0	2.0	326.4	2.9	1.4	167.2	17.1	984.7
2024_STR_T_R_H_CT_2	NM	210.6	<LOD	1.4	1.3	97.8	<LOQ	<LOD	63.3	11.1	385.4
2024_STR_T_R_H_CT_3	NM	12.3	<LOD	<LOD	<LOD	3.2	<LOD	<LOD	2.9	<LOD	18.4
2024_STR_T_R_H_CT_4	NM	56.4	<LOD	<LOD	<LOD	21.1	<LOD	<LOD	11.7	1.4	90.7
2024_STR_T_R_H_CT_5	NM	433.6	<LOD	1.6	1.2	133.4	1.3	<LOQ	65.0	8.2	644.5
2024_STR_T_R_H_CT_6	NM	44.3	<LOD	1.8	<LOQ	40.5	<LOQ	<LOD	18.3	3.5	108.3
2024_STR_T_R_H_CT_7	NM	893.4	<LOD	4.4	2.6	459.2	2.8	1.9	110.7	20.6	1495.6
2024_STR_T_R_H_CT_8	NM	266.0	<LOD	2.1	<LOQ	154.3	1.0	<LOQ	70.7	13.2	507.4
2024_STR_T_R_H_CT_9	NM	353.5	<LOD	1.6	1.1	210.4	1.5	1.1	109.8	15.5	694.5
2024_STR_T_R_H_CT_10	NM	79.9	<LOD	<LOD	<LOD	36.8	<LOD	<LOD	12.3	2.9	131.9
2024_STR_T_R_H_CT_11	NM	122.9	<LOD	1.2	<LOQ	144.6	<LOQ	<LOD	43.5	3.4	315.7
2024_STR_T_R_H_CT_12	NM	52.4	<LOD	<LOD	<LOD	101.5	<LOD	<LOD	13.2	2.8	169.9
2023STR_T_H_R_TC1	NM	136.6	<LOD	<LOD	<LOQ	116.7	<LOQ	1.0	51.0	7.4	312.9
2023STR_T_H_R_TC2	NM	123.9	<LOD	<LOD	<LOQ	123.2	1.2	1.1	57.3	8.0	314.6
2023STR_T_H_R_TC3	NM	140.0	<LOD	2.3	1.5	192.1	3.8	2.5	102.5	14.6	459.4
2023STR_T_H_R_TC4	NM	81.9	<LOD	<LOD	<LOD	39.3	<LOQ	<LOD	31.9	5.2	158.3
2023STR_T_H_R_TC5	NM	169.6	<LOD	<LOD	<LOQ	115.6	1.1	<LOQ	71.7	8.0	366.0
2023STR_T_H_R_TC6	NM	215.9	<LOD	<LOD	<LOD	88.1	<LOQ	1.0	39.0	5.2	349.2
2023_STR_T_H_R_MC1	MC-LR	<LOD	<LOD	<LOD	<LOD	20.9	<LOD	<LOD	<LOD	<LOD	20.9
2023_STR_T_H_R_MC2	MC-LR	<LOD	<LOD	<LOD	<LOD	6.2	<LOD	<LOD	<LOD	<LOD	6.2
2023_STR_T_H_R_MC3	MC-LR	<LOD	<LOD	<LOD	<LOD	7.7	<LOD	<LOD	<LOD	<LOD	7.7
2023_STR_T_H_R_MC4	MC-LR	<LOD	<LOD	<LOD	<LOD	4.0	<LOD	<LOD	<LOD	<LOD	4.0
2023_STR_T_H_R_MC5	MC-LR	<LOD	<LOD	<LOD	<LOD	4.6	<LOD	<LOD	<LOD	<LOD	4.6
2023_STR_T_H_R_MC6	MC-LR	<LOD	<LOD	<LOD	<LOD	11.1	<LOD	<LOD	<LOD	<LOD	11.1
2024_STR_T_R_H_MC_1	MC-LR	<LOD	<LOD	<LOD	<LOD	486.7	<LOD	<LOD	1.1	<LOD	487.8
2024_STR_T_R_H_MC_2	MC-LR	<LOD	<LOD	<LOD	<LOD	346.4	<LOD	<LOD	<LOD	<LOD	346.4
Overview of Microcystin concentrations in root samples (ng g^{-1}) (continued)											

Samples	Cultivation condition	MC-RR	NOD	MC-LA	MC-LF	MC-LR	MC-LY	MC-LW	MC-YR	MC-WR	Total MCs
2024_STR_T_R_H_MC_3	MC-LR	<LOD	<LOD	<LOD	<LOD	429.1	<LOD	<LOD	<LOD	<LOD	429.1
2024_STR_T_R_H_MC_4	MC-LR	<LOD	<LOD	<LOD	<LOD	67.4	<LOD	<LOD	<LOD	<LOD	67.4
2024_STR_T_R_H_MC_5	MC-LR	<LOD	<LOD	<LOD	<LOD	474.4	<LOD	<LOD	1.1	<LOD	475.5
2024_STR_T_R_H_MC_6	MC-LR	<LOD	<LOD	<LOD	<LOD	847.9	<LOD	<LOD	<LOD	<LOD	847.9
2024_STR_T_R_H_MC_7	MC-LR	<LOD	<LOD	<LOD	<LOD	734.8	<LOD	<LOD	1.4	<LOD	736.2
2024_STR_T_R_H_MC_8	MC-LR	<LOD	<LOD	<LOD	<LOD	1143.2	<LOD	<LOD	1.1	<LOD	1144.3
2024_STR_T_R_H_MC_9	MC-LR	<LOD	<LOD	<LOD	<LOD	222.6	<LOD	<LOD	<LOD	<LOD	222.6
2024_STR_T_R_H_MC_10	MC-LR	<LOD	<LOD	<LOD	<LOD	382.4	<LOD	<LOD	<LOD	<LOD	382.4
2024_STR_T_R_H_MC_11	MC-LR	<LOD	<LOD	<LOD	<LOD	1097.4	<LOD	<LOD	<LOQ	<LOD	1097.4

Table S3. Microcystin congeners concentration (ng g^{-1}) observed in the greens parts of strawberry plants after contamination with Hoagland solution fortified with either freeze-dried cyanobacterial biomass (NM) or pure MC-LR.

Accumulation of microcystin congeners in the green strawberry plant parts (ng g^{-1})											
Samples	Cultivation condition	MC-RR	NOD	MC_LA	MC-LF	MC-LR	MC-LY	MC-LW	MC-YR	MC-WR	Total MCs
2023STR_T_H_G_TC1	NM	1.3	<LOD	<LOD	<LOD	5.2	<LOD	<LOD	<LOQ	<LOD	5.2
2023STR_T_H_G_TC2	NM	<LOD	<LOD	<LOD	<LOD	7.5	<LOD	<LOD	2.2	<LOD	9.8
2023STR_T_H_G_TC3	NM	<LOD	<LOD	<LOD	<LOD	1.8	<LOD	<LOD	<LOD	<LOD	1.8
2023STR_T_H_G_TC4	NM	<LOD	<LOD	<LOD	<LOD	<LOD	<LOD	<LOD	<LOD	<LOD	0.0
2023STR_T_H_G_TC5	NM	<LOD	<LOD	<LOD	<LOD	8.6	<LOD	<LOD	2.8	<LOD	11.4
2023STR_T_H_G_TC6	NM	<LOD	<LOD	<LOD	<LOD	1.6	<LOD	<LOD	<LOD	<LOD	1.6
2024_STR_T_G_H_CT_1	NM	1.9	<LOD	<LOD	<LOD	<LOQ	<LOD	<LOD	<LOD	<LOD	1.9
2024_STR_T_G_H_CT_2	NM	3.2	<LOD	<LOD	<LOD	1.5	<LOD	<LOD	<LOD	<LOD	4.8
2024_STR_T_G_H_CT_3	NM	1.4	<LOD	<LOD	<LOD	1.5	<LOD	<LOD	<LOD	<LOD	2.9
2024_STR_T_G_H_CT_4	NM	<LOD	<LOD	<LOD	<LOD	<LOQ	<LOD	<LOD	<LOD	<LOD	0.0
2024_STR_T_G_H_CT_5	NM	2.6	<LOD	<LOD	<LOD	<LOQ	<LOD	<LOD	<LOD	<LOD	2.6
2024_STR_T_G_H_CT_6	NM	1.5	<LOD	<LOD	<LOD	<LOD	<LOD	<LOD	<LOD	<LOD	1.5
2024_STR_T_G_H_CT_7	NM	3.8	<LOD	<LOD	<LOD	1.5	<LOD	<LOD	<LOQ	<LOD	5.3
2024_STR_T_G_H_CT_8	NM	2.5	<LOD	<LOD	<LOD	1.0	<LOD	<LOD	<LOD	<LOD	3.5
2024_STR_T_G_H_CT_9	NM	2.2	<LOD	<LOD	<LOD	1.2	<LOD	<LOD	<LOD	<LOD	3.4
2024_STR_T_G_H_CT_10	NM	2.7	<LOD	<LOD	<LOD	1.3	<LOD	<LOD	<LOD	<LOD	4.0
2024_STR_T_G_H_CT_11	NM	<LOD	<LOD	<LOD	<LOQ	<LOQ	<LOD	<LOD	<LOD	<LOD	0.0
2024_STR_T_G_H_CT_12	NM	<LOD	<LOD	<LOD	1.4	1.1	<LOD	<LOD	<LOD	<LOD	2.5
2023_STR_T_G_H_MC_1	MC-LR	<LOD	<LOD	<LOD	<LOD	<LOD	<LOD	<LOD	<LOD	<LOD	
2023_STR_T_G_H_MC_2	MC-LR	<LOD	<LOD	<LOD	<LOD	<LOD	<LOD	<LOD	<LOD	<LOD	
2023_STR_T_G_H_MC_3	MC-LR	<LOD	<LOD	<LOD	<LOD	<LOD	<LOD	<LOD	<LOD	<LOD	
2023_STR_T_G_H_MC_4	MC-LR	<LOD	<LOD	<LOD	<LOD	<LOD	<LOD	<LOD	<LOD	<LOD	
2023_STR_T_G_H_MC_5	MC-LR	<LOD	<LOD	<LOD	<LOD	<LOD	<LOD	<LOD	<LOD	<LOD	
2023_STR_T_G_H_MC_6	MC-LR	<LOD	<LOD	<LOD	<LOD	<LOD	<LOD	<LOD	<LOD	<LOD	
2023_STR_T_G_H_MC_7	MC-LR	<LOD	<LOD	<LOD	<LOD	<LOD	<LOD	<LOD	<LOD	<LOD	
2023_STR_T_G_H_MC_8	MC-LR	<LOD	<LOD	<LOD	<LOD	<LOD	<LOD	<LOD	<LOD	<LOD	

Accumulation of microcystin congeners in the green strawberry plant parts (ng g-1)											
Samples	Cultivation condition	MC-RR	NOD	MC_LA	MC-LF	MC-LR	MC-LY	MC-LW	MC-YR	MC-WR	Total MCs
2024_STR_T_G_H_MC_1	MC-LR	<LOD	<LOD	<LOD	<LOD	3.0	<LOD	<LOD	<LOD	<LOD	3.0
2024_STR_T_G_H_MC_2	MC-LR	<LOD	<LOD	<LOD	<LOD	1.1	<LOD	<LOD	<LOD	<LOD	1.1
2024_STR_T_G_H_MC_3	MC-LR	<LOD	<LOD	<LOD	<LOD	2.7	<LOD	<LOD	<LOD	<LOD	2.7
2024_STR_T_G_H_MC_4	MC-LR	<LOD	<LOD	<LOD	<LOD	<LOD	<LOD	<LOD	<LOD	<LOD	0.0
2024_STR_T_G_H_MC_5	MC-LR	<LOD	<LOD	<LOD	<LOD	1.6	<LOD	<LOD	<LOD	<LOD	1.6
2024_STR_T_G_H_MC_6	MC-LR	<LOD	<LOD	<LOD	<LOD	1.3	<LOD	<LOD	<LOD	<LOD	1.3
2024_STR_T_G_H_MC_7	MC-LR	<LOD	<LOD	<LOD	<LOD	1.6	<LOD	<LOD	<LOD	<LOD	1.6
2024_STR_T_G_H_MC_8	MC-LR	<LOD	<LOD	<LOD	<LOD	2.3	<LOD	<LOD	<LOD	<LOD	2.3
2024_STR_T_G_H_MC_9	MC-LR	<LOD	<LOD	<LOD	<LOD	4.2	<LOD	<LOD	<LOD	<LOD	4.2
2024_STR_T_G_H_MC_10	MC-LR	<LOD	<LOD	<LOD	<LOD	<LOQ	<LOD	<LOD	<LOD	<LOD	0.0
2024_STR_T_G_H_MC_11	MC-LR	<LOD	<LOD	<LOD	<LOD	12.7	<LOD	<LOD	<LOD	<LOD	12.7
